# Stable glenoid component of reverse total shoulder arthroplasty at 2 years as measured with model-based radiostereometric analysis (RSA)

**DOI:** 10.1080/17453674.2021.1943932

**Published:** 2021-07-01

**Authors:** Alexander Nilsskog Fraser, Berte Bøe, Tore Fjalestad, Jan Erik Madsen, Stephan M Röhrl

**Affiliations:** a Division of Orthopaedic Surgery, Oslo University Hospital; b Institute of Clinical Medicine; University of Oslo; c Diakonhjemmet Hospital,Norway

## Abstract

Background and purpose — Reverse total shoulder arthroplasty (TSA) is used for treating cuff arthropathy, displaced proximal humeral fractures (PHF), and in revision shoulder surgery, despite sparse evidence on long-term results. We assessed stability of the glenoid component in reverse TSA, using model-based RSA.

Patients and methods — 20 patients (mean age 76 years, 17 female), operated on with reverse TSA at Oslo University Hospital, in 2015–2017 were included. Indications for surgeries were PHFs, malunion, cuff arthropathy, and chronic shoulder dislocation. RSA markers were placed in the scapular neck, the coracoid, and the acromion. RSA radiographs were conducted postoperatively, at 3 months, 1 year, and 2 years. RSA analysis was performed using RSAcore with Reversed Engineering (RE) modality, with clinical precision < 0.25 mm for all translations (x, y, z) and < 0.7° for rotations (x, z). Scapular “notching” was assessed in conventional radiographs.

Results — 1 patient was excluded due to revision surgery. More than half of the patients displayed measurable migration at 2 years: 6 patients with linear translations below 1 mm and 8 patients who showed rotational migration. Except for one outlier, the measured rotations were below 2°. The migration pattern suggested implant stability at 2 years. 10 patients showed radiolographic signs of “notching”, and the mean Oxford Shoulder Score (OSS) at 2 years was 29 points (15–36 points).

Interpretation — Stability analysis of the glenoid component of reversed total shoulder arthroplasty using reversed engineering (RE) model-based RSA indicated component stability at 2 years.

Reverse total shoulder arthroplasty (TSA) is a widely used procedure. It was originally intended for cuff arthropathy in elderly patients (Grammont and Baulot 1993), but is presently used for several indications, including acute proximal humeral fractures (PHFs) in the elderly, fracture malunions, chronic dislocations, and revision surgery (Clavert et al. 2019, Rugg et al. 2019, Malahias et al. 2020). For operative treatment of displaced 3- and 4-part PHFs in the elderly, reversed TSA has become the treatment of choice (Critchley et al. 2020), presently down to 60 years of age (Goldenberg et al. 2020).

The increased use of reverse TSA has occurred despite sparse evidence concerning long-term clinical outcomes for the implant. However, short-term RSA may predict the longevity of implants (Valstar et al. 2005). For hips and knees, continuous micro-migration over 2 years has shown to be indicative of increased risk of implant loosening (Kärrholm et al. 1994, de Vries et al. 2014). To our knowledge, RSA stability analysis of the glenoid component of reverse TSA in patients has not previously been published.

Much concern has been placed on the subject of “notching,” where the polyethylene liner of a reverse TSA over time erodes into the inferior scapular neck (Levigne et al. 2011). Several studies have related notching to poorer outcomes (Mollon et al. 2017, Simovitch et al. 2019), while others have voiced concerns about this causing instability and loosening of the glenoid component (Roche et al. 2013c, Huri et al. 2016).

Model-based RSA has the advantage over traditional marker-based RSA of not having to alter implants by attaching markers, and the clinical precision of model-based RSA on the glenoid component is known (Fraser et al. 2018). With increased use, sparse long-term evidence, and with “notching” as the backdrop, we performed a stability analysis of the glenoid component of reversed TSA, using model-based RSA.

## Patients and methods

In this clinical RSA study of the glenoid component of reverse TSA we have used model-based RSA technology, and specifically the reversed engineering (RE) RSA modality. A published method study (Fraser et al. 2018) supplied the framework, particularly with regard to how patients were positioned for RSA examinations, which RSA software modality was used, and the clinical precision of the RE model-based RSA on this implant, obtained by double examinations of 15 patients included in the current study.

### Inclusion

20 consecutive patients operated on with reverse TSA at Oslo University Hospital in the period from September 2015 to October 2017 were included. The majority of included patients had suffered an acute PHF, and a minority of patients were included on the basis of other indications (Table 1). Initially, we planned to include only fracture patients enrolled in a larger clinical trial comparing reverse TSA with plate fixation for displaced PHFs in the elderly (Fraser et al. 2020). The original inclusion criteria were patients aged 65–85 presenting with a displaced PHF type 11-B2 or 11-C2 (OTA/AO 2007 revision). All subgroups of B2 and C2 fractures were included, provided that the fractures were severely displaced, defined as > 45° valgus or > 30° varus in a true antero-posterior (AP) projection, > 45° angulation in the scapula Y-projection, or > 50% displacement of the humeral head against the metaphysis. Exclusion criteria were previous injury or illness of the injured or contralateral shoulder, concomitant injury to the ipsilateral or contralateral upper extremity, alcohol or other substance abuse, dementia or neurological disease, non-Norwegian speaking, glenoid fracture or deformity, or patients who were deemed non-compliant with rehabilitation. Head-split fractures or fracture dislocations were not included. These criteria resulted in a slow inclusion rate, and we therefore changed the inclusion criteria to any patient destined for reverse TSA at Oslo University Hospital.

**Table 1.** Baseline demographics in the 20 included patients. Values are count unless otherwise specified

Sex (male/female)	3/17
Mean age (SD)	76 (5.7)
Median age (range)	77 (66–85)
Living at home	20
Diabetes (yes/no)	2/18
Smoking (yes/no)	4/16
Mean ASA group (SD)	2.2 (0.4)
Operated arm (right/left)	8/12
Indication for surgery	
Acute PHF	13
Malunion PHF	1
Delayed surgery PHF	2
Cuff-tear arthropathy	3
GH luxation (chronic)	1
Oxford Shoulder Score (n = 5)	35

### Operative treatment

**Figure 1.**
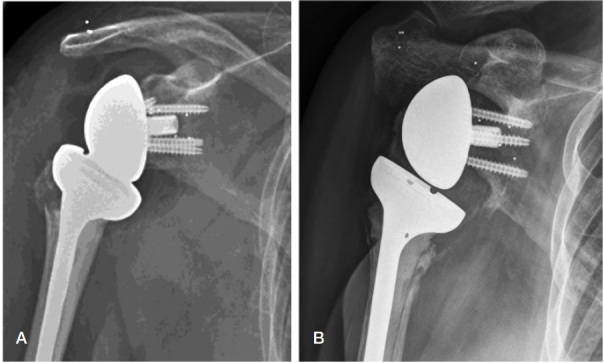
Radiograph of a patient operated with a reverse total shoulder arthroplasty postoperatively (A) and at 2-year follow-up (B), where “notching” grade 3 can be observed in the inferior scapular neck. RSA markers were implanted in the scapular neck, coracoid, and acromion.

Operative treatment with a reversed TSA (Delta Xtend Depuy Synthes, 700 Orthopaedic Drive, Warsaw, IN 46582, USA) was performed with all patients in the beach-chair position. In the 16 patients with PHF, acute or delayed, and 1 patient with a chronic glenohumeral dislocation, a deltopectoral approach was used. In the 3 patients with cuff arthropathy, a lateral transdeltoid approach was used. The tantalum markers were implanted in the glenoid, the acromion, and the coracoid process after surgical preparation of the glenoid, before implant insertion (Figure 1). Approximately 10 markers were used for each patient, adding about 15 minutes to the overall surgical procedure.

### RSA radiographs

**Figure 2.**
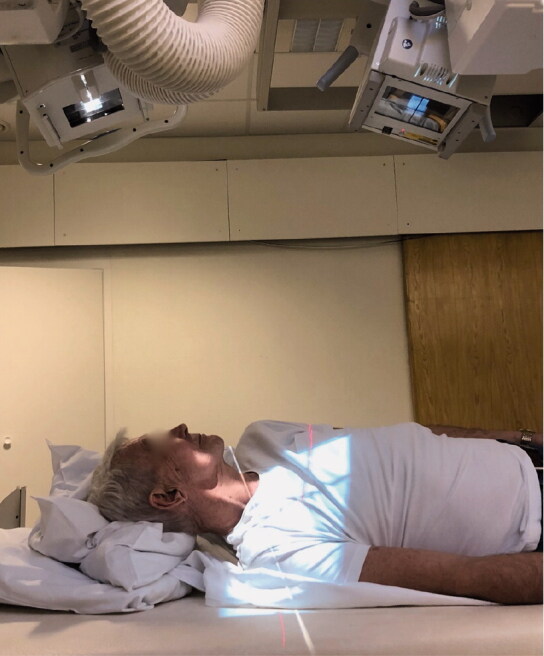
Patient positioning when performing shoulder RSA. The radiographic tubes were centered on the glenoid implant of the right shoulder and aligned along the length axis of the patient, resulting in a sagittal radiographic plane.

Paired RSA radiographs were obtained postoperatively (PO), at 3 months, 1 year, and 2 years. We strived to conduct PO radiographs within 1 week, and most PO examinations were performed on day 3 or 4 after surgery. RSA examinations were dual simultaneous radiographs, where the overhead X-ray tubes were focused on the implant at a mutual angle of 60°. The patient was positioned supine on the examination table, and approximately 20–30° tilted towards the operated side, with radiographic exposure in the sagittal plane. We named this the shoulder position (Figure 2) to distinguish it from traditional hip RSA where the radiographic plane is transverse. A uniplanar calibration cage (Cage 43, UmRSA Biomedical—RSA Biomedical, Umeå, Sweden) was positioned underneath the examination table.

### Reference position

We decided that the right shoulder with the peg of the glenoid implant pointing upwards on radiographs would be the reference position for RSA migration analyses. The clinical significance and direction of translation and rotation are listed on the left side of Table 2. For any left-sided implant, or for radiographs with the peg of the glenoid implant pointing downwards, certain conversions of direction would have to be applied before conducting the final migration analysis. These conversions are listed on the right side of Table 2, and basically convert all migration values to the decided reference position: right shoulder with peg pointing upwards.

**Table 2.** Shoulder reference position and conversion table

	Right shoulder: Clinical direction of migration/rotation	Left shoulder: Conversion to right shoulder values	Peg down: Conversion to peg up
Translation			
x	cranial (–), caudal (+)	Ч (–1)	Ч (–1)
y	lateral (–), medial (+)	No difference	Ч (–1)
z	posterior (–), anterior (+)	No difference	No difference
Rotation			
x	internal (–), external (+)	No difference	Ч (–1)
y	NP	NP	NP
z	superior tilt (–),inferior tilt (+)	Ч (–1)	No difference

The right shoulder with the peg up is the reference position, and the clinical significance of a negative or positive value for all translations and rotations are listed on the left side of this table. For the purpose of RSA migration analyses, left-sided implants or implants that are orientated with the peg down on RSA radiographs, must be converted to the reference position, as described on the right side of the table. NP = Not possible to analyze.

### RSA analysis

**Figure 3.**
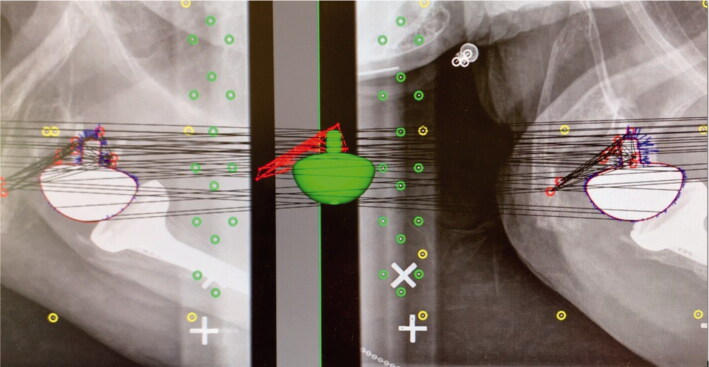
Radiostereometric analyses were performed with RSAcore software using reversed engineering (RE). The virtual glenoid implant (green) corresponds with the red demarcated outline of the actual glenoid implant shown on dual simultaneous radiographs of a right-sided shoulder with a reverse total shoulder arthroplasty. RSA markers are red, control markers are green, and fiducial markers are yellow.

RSA radiographs were analyzed using RSAcore software (MB-RSA 4.1, Leiden University Medical Centre, Leiden, NL), using reversed engineering (RE) (Figure 3). The RE model of the glenoid implant (Delta Xtend Metaglene and Glenosphere, Depuy Synthes, 700 Orthopaedic Drive, Warsaw, IN 46582, USA) was obtained by laser-scanning (RSAcore, Leiden University Medical Centre, Leiden, NL). The circumference of the glenoid component in the paired radiographs was matched with the virtual RE model of the implant. The RSA markers implanted in scapular bone were marked manually. Migration of the implant along each of the 3 axes (x, y, z) and rotation around the 2 measurable axes (x and z) were measured from point 0, corresponding with PO radiographs, to 2-year follow-up. The distribution of RSA bone markers was assessed using the condition number (CN) (Valstar et al. 2005). RSA radiographs with a CN > 120, indicating a narrow distribution of markers, were omitted. RSA radiographs with a CN < 120, indicating a wider distribution of markers, were included. The mean error for rigid body fitting (ME) (Valstar et al. 2005), is an expression of marker stability, where the limit in this study was set to ME < 0.35. Any marker presenting with a ME > 0.35 would imply that the marker was unstable, and was therefore omitted.

### Clinical outcome

Oxford Shoulder Score (OSS, 0–48 points, 0 = worst, 48 = best) was assessed at 2 years. Scapular notching was assessed on conventional shoulder radiographs obtained in conjunction with clinical follow-ups.

### Statistics

The mean value with a confidence interval (CI) of ± 2 standard deviations (± 2SD) for linear migration and rotation was calculated separately for each degree of freedom. Statistical analyses were performed using IBM SPSS Statistics Version 24 (IBM Corp, Armonk, NY, USA).

### Ethics, funding, and potential conflicts of interest

Ethical approval was obtained from the regional ethics committee (REK) on April 4, 2015, reference No. 2012/1606/REK South-East, and patients gave signed consent after written and oral information. The project has received research funds from Sophies Minde Ortopedi AS, a subsidiary of Oslo University Hospital. The authors have no conflict of interest to declare.

## Results

**Figure 4.**
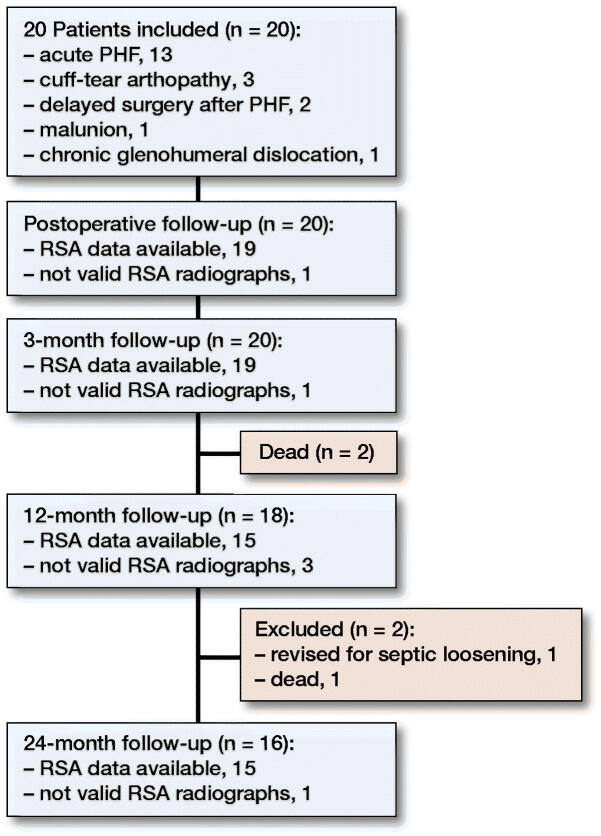
Flow of patients.

20 patients with a mean age of 76 (66–85) years were included, all living at home. All patients were treated operatively with reverse TSA, and in 15 of 20 cases the indication was a displaced proximal humeral fracture (Table 1). 1 patient was excluded from the final analysis because of septic implant loosening and revision surgery, 3 patients died before two-year follow-up, and 1 patient was excluded from 24-month RSA analysis because of CN > 120 (Figure 4).

**Figure 5.**
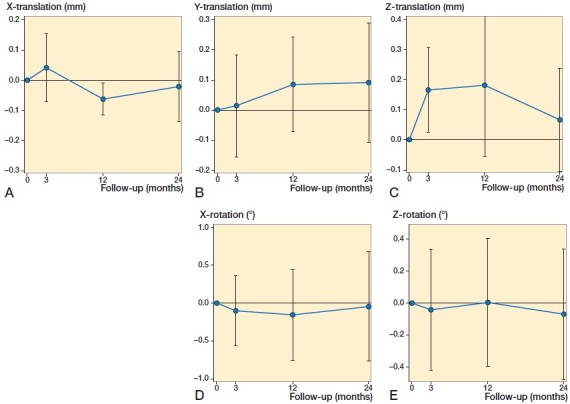
Mean values for (A) x-translations (negative values = cranial migration, positive values = caudal migration), (B) Y-translations (negative values = lateral migration, positive values = medial migration), (C) Z-translations (negative values = posterior migration, positive values = anterior migration) measured in mm, and (D) X-rotation (negative values = internal rotation, positive values = external rotation) and (E) Z-rotation (negative values = superior tilt, positive values = inferior tilt) measured in degrees.

Mean migration measurements showed linear translations along the x, y, and z axis, signifying caudal–cranial, lateral–medial and anterior–posterior translations respectively. Rotational measurements around the x-axis and z-axis signified internal–external rotation and superior–inferior tilt of the implant (Figure 5). Rotation around the y-axis (anterior–posterior flexion) is not possible to measure with model-based RSA, as this implant is symmetrical around this axis.

**Figure 6.**
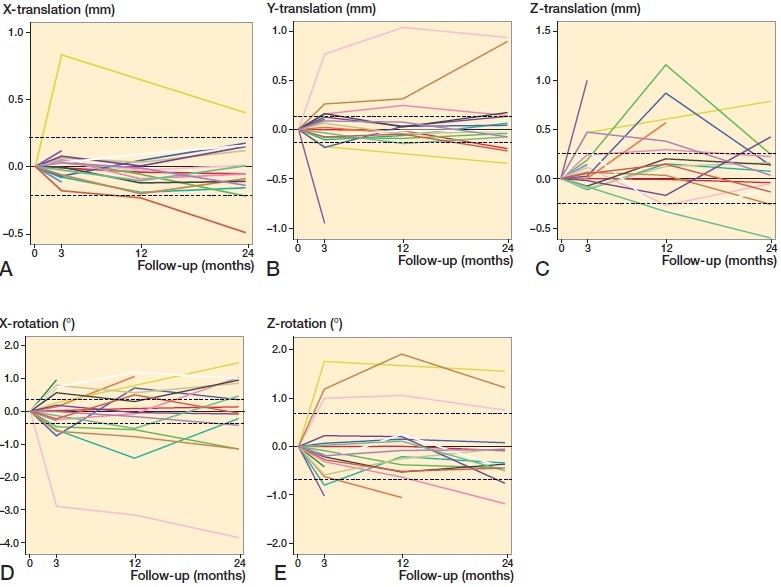
Individual patient RSA measurements for translations and rotations. For directions (A–E), see Figure 5. Stippled horizontal lines define the clinical precision of reverse-engineered model-based RSA on the glenoid component, where the area between the two stippled lines represent migration that is outside the resolution of this RSA method.

Individual migration measurements for each patient were similarly divided into linear translations and rotations, where the precision of the RSA method for each degree of freedom was marked with two stapled lines (Fraser et al. 2018) (Figure 6). Of the 19 patients included in the final analysis, 17 patients demonstrated measurable translation and/or rotation above the clinical precision of RSA on the glenoid component. Of these, no linear migrations exceeded 1.2 mm in any direction, and apart from 1 outlier (No. 8: internal rotation), the rotations measured were below 2°.

2 patients had a grade 1 notching, with a defect contained within the inferior pillar of the scapular neck; 5 patients had grade 2 notching where the erosion had reached the inferior screw; and 2 patients had grade 3 notching with erosion of bone beyond the lower fixation screw. The mean OSS at 2 years was 29 points (15–36). Of the 15 patients with acceptable RSA radiographs at 2 years, 9 patients displayed radiographic notching, all of which showed measurable migration in 1 or more degrees of freedom. 6 patients did not show signs of notching at 2 years (Table 3).

**Table 3.** Individual patient data at 2 years

Patient no.	Indication	Notching grade	OSS	Dropout
1	Malunion			Dead
2	Acute PHF		33	
3	Acute PHF			Dead
4	Late PHF			Dead
5	Late PHF	2		Revised
6	Acute PHF	3	35	
7	Acute PHF		24	
8	Arthrosis	1	29	
9	Chronic			
	dislocation		16	
10	Acute PHF		21	
11	Arthrosis	2	35	
12	Acute PHF		33	
13	Arthrosis	3	36	
14	Acute PHF	2	33	
15	Acute PHF		28	
16	Acute PHF	2	36	
17	Acute PHF	2	31	
18	Acute PHF	1	15	
19	Acute PHF	2	26	
20	Acute PHF		36	

OSS = Oxford Shoulder Score,

PHF = proximal humeral fracture.

## Discussion

Our study of model-based RSA on the glenoid component of reverse TSA has shown that approximately half of the patients displayed migration below the precision level of the RSA method. With the exception of 1 outlier, which displayed approximately 4° internal rotation at 2 years, the others had a measurable migration up to ∼1 mm translation or 2° rotation over 2 years. When considering the individual RSA migration measurements (Figure 6), all lines—including those that present early measurable migration—seem to conform towards the horizontal. This migration pattern indicates a stable implant.

Little RSA research has been published on this implant. Apart from a methodological study including precision measurements in patients (Fraser et al. 2018), our study seems to be the only clinical RSA stability study on reverse shoulder arthroplasty. Other shoulder RSA studies have involved a phantom glenoid component (Van de Kleut et al. 2018), anatomic shoulder arthroplasties or resurfacing implants (Nagels et al. 2002, Rahme et al. 2004, 2006, 2009, Nuttall et al. 2009, 2012, Sköldenberg and Odquist 2011, Stilling et al. 2012, Mechlenburg et al. 2014, Streit et al. 2015). These latter studies utilized marker-based RSA, and differ fundamentally from the current study with regard to implant design, fixation type, and RSA method. Comparisons therefore seem futile, and may even be misleading.

An association between early migration and later implant loosening has not been established for reversed shoulder arthroplasties. For the hip, Pijls et al. (2012) conducted a systematic review to determine the association between early migration of acetabular cups and late aseptic revision, where proximal migration of < 0.2 mm was considered acceptable, while proximal migration > 1mm was considered unacceptable. A proximal migration of between 0.2 mm and 1.0 mm at 2 years carried a > 5% risk of revision at 10 years.

In the context of model-based RSA, acetabular cups bear some resemblance to the glenoid implants in reverse TSA, in the sense that they are both small implants with a hemispherical shape. Even so, these implants are not readily comparable. Hip implants are subjected to different forces and weight-bearing, and the acetabular cups in this study had a variety of fixation modalities, including cement fixation. Despite the obvious differences, it would be interesting to compare the thresholds (Pijls and Nelissen 2016) for increased risk of loosening.

In our study, 13 of 15 patients were within ±0.22 mm caudal-cranial migration at 2 years, while 2 patients show measurable migration of less than 0.5 mm (Figure 6A). At 2 years, these patients had an OSS score of 35 and 26 points, and displayed notching grade 3 and 2 (Table 3). Whether a migration of 0.5 mm combined with notching represents an increased risk of implant loosening remains to be seen, and long-term follow-up is needed to establish threshold migration values for the glenoid component. Further, these thresholds may be compared with established threshold values of other implants.

Conventional radiographic projections were obtained at every follow-up, and these were used to detect glenoid notching. 9 patients had signs of notching on 2-year AP radiographs: 6 with acute PHF and 3 with cuff arthropathy. This constituted half of the patients included in this study, and a higher fraction than expected, especially due to the fact that the operative technique has been altered to prevent this from happening (Roche et al. 2013a, 2013b). Except for 1 patient, all patients with notching displayed measurable migration in at least 1 degree of freedom (Table 3).

The mean OSS was 29 points, which is substantially lower than the reverse TSA group in the DelPhi study, an RCT comparing reverse TSA with plate fixation for displaced PHFs (Fraser et al. 2020), where they scored 41 points at 2 years. However, the heterogenicity of indications in our study is a limitation, and makes OSS comparisons with other studies difficult. Besides acute PHFs, our study involved patients with operative indications that may have worsened the outcome, such as chronic dislocation, malunion, PHF delayed surgery, and cuff arthropathy. Furthermore, half the patients in our study showed radiological signs of notching, which is known to be associated with poorer outcomes (Mollon et al. 2017, Simovitch et al. 2019).

20 patients is a sparse number for most clinical trials, but arguably sufficient for an RSA study due to the high precision of the method (Valstar et al. 2005). Another limitation of our study was the variety of indications for reverse TSA. This makes some of the results more difficult to compare with other studies. Heterogenicity of indications, however, does not affect the overall result of what would be described as a stable glenoid component migration pattern.

Furthermore, rotation around the y-axis, representing anteversion/retroversion of the glenoid implant, was not measurable with model-based RSA due to the implant being symmetrical around this axis. This also implied that maximal total point motion (MTPM) could not be calculated.

One strength of our study was using an established RSA method previously tested in a methodological study (Fraser et al. 2018), where patient positioning, type of model-based RSA, and the clinical precision of the RSA method was established in advance.

In conclusion, stability analysis of the glenoid component of reversed total shoulder arthroplasty using reversed engineering (RE) model-based RSA indicate component stability at 2 years.
